# Transanal versus Laparoscopic Total Mesorectal Excision in Male Patients with Low Tumor Location after Neoadjuvant Therapy: A Propensity Score-Matched Cohort Study

**DOI:** 10.1155/2022/2387464

**Published:** 2022-02-27

**Authors:** Ze Li, Jingkun Xiao, Yujie Hou, Xingwei Zhang, Haiqing Jie, Huashan Liu, Lei Ruan, Ziwei Zeng, Liang Kang

**Affiliations:** ^1^Department of Colorectal Surgery, The Sixth Affiliated Hospital of Sun Yat-sen University, Guangzhou, Guangdong 510655, China; ^2^Guangdong Institute of Gastroenterology, The Sixth Affiliated Hospital of Sun Yat-sen University, Guangzhou, Guangdong 510655, China; ^3^Guangdong Provincial Key Laboratory of Colorectal and Pelvic Floor Diseases, The Sixth Affiliated Hospital of Sun Yat-sen University, Guangzhou, Guangdong 510655, China

## Abstract

**Background:**

Since Sylla and Lacy successfully reported the transanal total mesorectal excision in 2010, taTME was considered to have the potential to overcome some problematic laparoscopic cases in male, low advanced rectal cancer. However, the evidence is still lacking. This study compared the short and long outcomes of taTME with laTME in these “challenging” patients to explore the advantages of taTME among the patients.

**Method:**

After propensity score matching analysis, 106 patients were included in each group from 325 patients who met the including standard. Statistical analysis was used to compare the differences of perioperative outcomes, histopathological results, and survival results between taTME and laTME groups.

**Results:**

The mean time of pelvic operation in the taTME group was significantly shorter than in the laTME group (62.2 ± 14.2 mins vs 81.1 ± 18.9 mins, *P* = 0.003). The complication incidence rate and the rate of protective loop ileostomy in the taTME group were significantly lower than those in the laTME group (19.8% vs 38.7%, *P* = 0.003 and 70.8% vs 92.5%, *P* < 0.001). In long-term result, there was no significant difference between the two groups for 3-year OS (87.3% vs 85.4%, *P* = 0.86) or 3-year DFS (74.9% vs 70.1%, *P* = 0.92). The 2-year cumulative local recurrence rate was similar between the two groups (1.1% vs 5.8%, *P* = 0.22).

**Conclusion:**

This study demonstrated that taTME might reduce the incidence of postoperative complications, especially of anastomotic leakage in these “challenging” patients. taTME may be considered to have clear advantages for “challenging” patients.

## 1. Introduction

Since the concept of total mesorectum excision (TME) was described by Heald in 1979 [1], it has become a gold standard for rectal cancer surgery. According to the results of published randomized clinical trials (RCT) [2, 3], laparoscopic TME (laTME) is considered an effective alternative method to open surgery and has become the mainstream treatment for mid and low rectal cancer. Although laTME has been associated with better visualization in the pelvic cavity than open surgery, it remains challenging to perform operations in a narrow and deep space to obtain high-quality resected specimens due to fixed trocar positions and straight laparoscopic instruments [4].

Since transanal total mesorectal excision (taTME) was pioneered by Sylla et al. [5], it has become a hot topic in treating middle to low rectal cancer. In comparison to laTME, taTME has a “down-to-up” and “inner-to-outer” approach. The procedure can achieve an accurate distal resection margin with great visualization during surgery [6]. Thus, it is considered a method to solve the “old problem” [7], especially in male rectal cancer patients with low tumor position or those who underwent neoadjuvant therapy. Herein, we define a patient featured by all of the above conditions as a “challenging” patient. In such patients, sufficient free space cannot be obtained when dissociating from head-to-tail due to more curved sacrum and pelvic tissue edema [8]. Furthermore, “challenging” patients not only present a higher risk of complications during laparoscopic surgery but also feature undesired mesorectal resection quality and status of the resection margin, which may lead to poor prognosis such as local recurrence [9]. While the “down-to-up” approach in taTME has been considered to largely overcome these difficult aspects of open surgery or laparoscopic surgery [7], studies reporting taTME results for “challenging” patients are still lacking. Therefore, the specific aim of the present study was to explore the advantages of taTME among “challenging” patients by comparing the outcomes of taTME with laTME performed in such patients.

## 2. Methods

### 2.1. Patients

All consecutive clinical records of patients who underwent taTME performed by experienced surgeons with two groups in the Sixth Affiliated Hospital of Sun Yat-sen University (Guangzhou, China) from July 2014 to July 2020 were reviewed. The inclusion criteria were as follows: (1) male, (2) history of neoadjuvant therapy, and (3) tumor location < 5 cm from the anal margin. Female patients, underwent emergency surgery or abdominoperineal resection, were excluded from this study. Subsequently, this cohort was matched with patients who underwent laTME performed by experienced surgeons in our center by propensity score matching (PSM), which can minimize selection bias caused by the retrospective analysis [10, 11].

Baseline characteristics were regarded as the covariates that may impact selection bias in the retrospective analysis, including age, body mass index (BMI), preoperative incomplete intestinal obstruction, history of past abdominal surgery, tumor location, tumor size, tumor stage, and type of neoadjuvant therapy regimen.

### 2.2. Measured Outcomes

Data were collected in specific tables by referring to medical records and the institutional prospective colorectal cancer database. The baseline demographic and clinical data were the same as the covariates. For preoperative staging, enhanced CT and MR imaging (or endorectal ultrasound) have been used. If the patient underwent neoadjuvant radiotherapy, the surgery was planned for a minimum of 8 weeks after the last radiotherapy.

**Figure 1:**
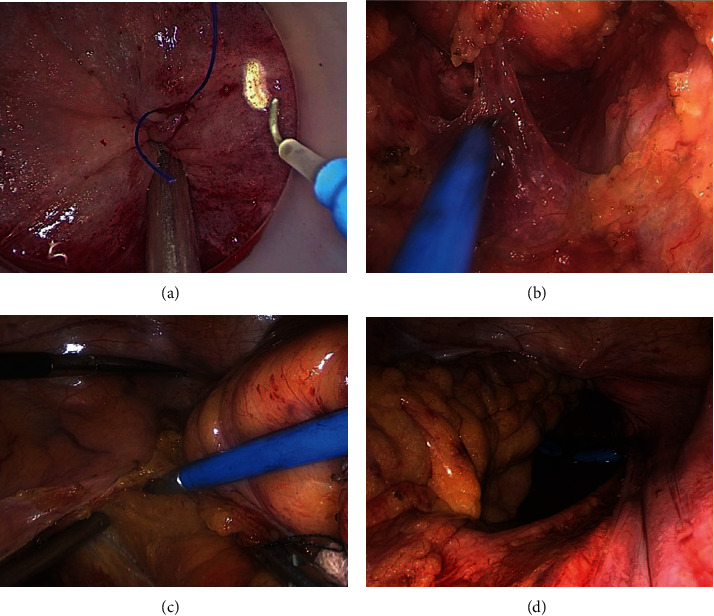
(a) Beginning of the purse-string suture. (b) Completion of TME by the opening of the peritoneum reflection. (c) Incision peritoneal reflection on the anterior lateral sided. (d) After cutting off the bowel at the distal tumor edge.

Perioperative outcomes are defined as surgery-related outcomes, including total operative time, pelvis operative time, intraoperative blood loss, conversion rate, protective loop ileostomy rate, postoperative complication, postoperative hospital stay, and 30-day postoperative mortality. The Clavien-Dindo classification was used to categorize the early postoperative complications [12]. In this study, pelvic operative time in the taTME group was defined as the time from the beginning of the purse-string suture (Figure 1(a)) to the completion of TME by opening the peritoneum reflection (Figure 1(b)). Meanwhile, the time in the laTME group was from the incision peritoneal reflection on the anterior lateral side (Figure 1(c)) or the separation of the S2 level on the posterior side to the plane of the levator anus muscle, to cutting off the bowel at the distal tumor edge (Figure 1(d)). Protective loop ileostomy depends on the blood supply and edema of the anastomotic stoma. Within the first 30 days following surgery, any clinical or radiological evidence of a defect of the anastomotic stoma was considered an anastomotic leak. All patients get an MRI in regular follow-up.

Pathological outcomes include the quality of the mesorectal specimen, the distance between tumor and distal resection margin, distal resection margin (DRM) status, number of harvested lymph nodes, and circumferential resection margin (CRM) involvement. A negative radial resection margin was defined as greater than 1 mm distance between the tumor or malignant lymph node and CRM [13, 14]. The quality of the mesorectum specimen was classified into three grades: (i) incomplete, (ii) nearly complete, and (iii) complete [15]. Tumors were staged by the TNM classification (8th edition) [16].

### 2.3. Statistical Analysis

All analyses *were carried out* using the Statistical Package for the Social Sciences Software (v26.0; SPSS Inc, Chicago, IL, USA). First, the normal distribution of continuous variables was tested by the Shapiro-Wilk test. Next, the normal distribution of continuous variables was described as mean ± standard deviation (range) and analyzed using the independent samples *t*-test. The abnormal distribution of continuous variables was presented as median (range) and analyzed using the Mann–Whitney *U* test. Categorical variables were reported as the number of patients (percentage) and compared by the *χ*2 test or the Fisher's exact test. A *P* value of less than 0.05 was defined as statistically significant. A propensity score was calculated by a logistic regression model for the variables shown in Table 1. Propensity score matching was then used with a 1 : 1 nearest neighbor matching algorithm. Unmatched patients were excluded. A caliper distance of 0.1 of the pooled standard deviation of the logit of the propensity score was applied.

## 3. Results

**Figure 2:**
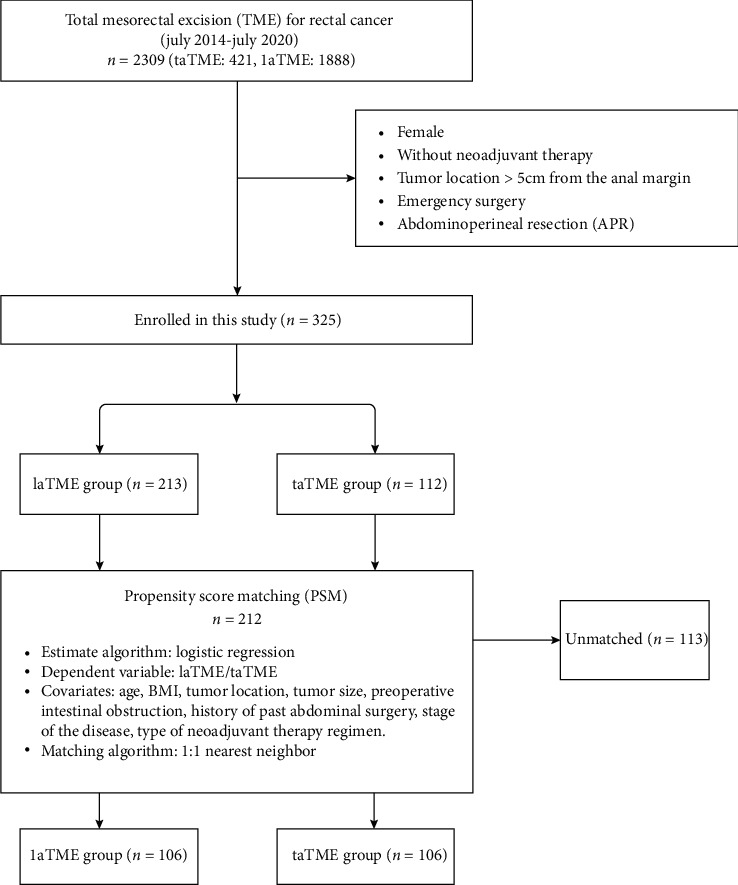
Flow chart of patient selection.

Among all 2309 patients, 325 patients met the set standard, including 213 in the laTME group and 112 in the taTME group. Eventually, 106 patients were included per group for further analysis after PSM (Figure 2).

### 3.1. Prematching and Postmatching of Baseline Characteristics

The details of the 1 : 1 PSM process are shown in Figure 2. After matching, demographic and clinical data between the two groups became more balanced, as described in Table 1. Moreover, no significant differences were shown between the two groups regarding the baseline demographic and clinical data.

### 3.2. Perioperative Outcomes

Results on perioperative outcomes are summarized in Table 2. No intraoperative complications occurred in either group. The average pelvis operative time was significantly less in the taTME group as compared with the laTME group (62.2 ± 14.2 min vs 81.1 ± 18.9 min, *P* = 0.003). The mean intraoperative bleeding loss in the taTME group was less than that in the laTME group (90 ± 156 mL vs 103 ± 136 mL, *P* = 0.004). The protective loop ileostomy rate was significantly lower in the taTME group (70.8% vs 92.5%, *P* < 0.001). In addition, the mean distance between anastomosis and the anal margin was larger in this group (2.3 ± 1.0 cm vs 2.0 ± 1.1 cm, *P* = 0.051). It is worth noting that there were three cases of conversion to taTME and open surgery in the laTME group. Two of these cases were converted to taTME because the pelvic stenosis resulted in an unclear surgical field and excessive bleeding that could not continue the separation. In a further case, open surgery was opted for due to inadequate blood supply of the proximal intestinal tube during anastomosis and the difficulty in redissociating the splenic flexure. The overall rate of postoperative complications was significantly lower in the taTME group than in the laTME group (19.8% vs 38.7%, *P* = 0.003), especially the anastomotic leakage rate (14.2% vs 25.2%, *P* = 0.017). Moreover, the median hospital stay after taTME was one day shorter than after laTME (9 days vs 10 days, *P* < 0.001).

### 3.3. Pathological Results

Detailed data obtained on histopathological outcomes are depicted in Table 3. According to histopathological results, the majority of patients had T3 or N0 lesions. In the taTME group, the mean distance between the inferior tumor and the distal margin was shorter (1.2 ± 0.9 cm) compared with the laTME group (1.3 ± 1.9 cm). However, the difference did not reach statistical significance (*P* = 0.394). In addition, the DRM was positive in one case (1.5%) in each group. No patients had positive CRM in the taTME group, while two patients (3.0%) in the laTME group presented positive CRM (*P* = 0.498). These two patients both had T4 lesions, were featured by the preoperative staging of T3, and underwent neoadjuvant radiochemotherapy treatment. In terms of mesorectal specimen quality, even though the complete resection rate in the taTME group was higher than that in the laTME group, the difference was not statistically significant (95.3% vs 91.5%, *P* = 0.269).

### 3.4. Follow-Up

**Figure 3:**
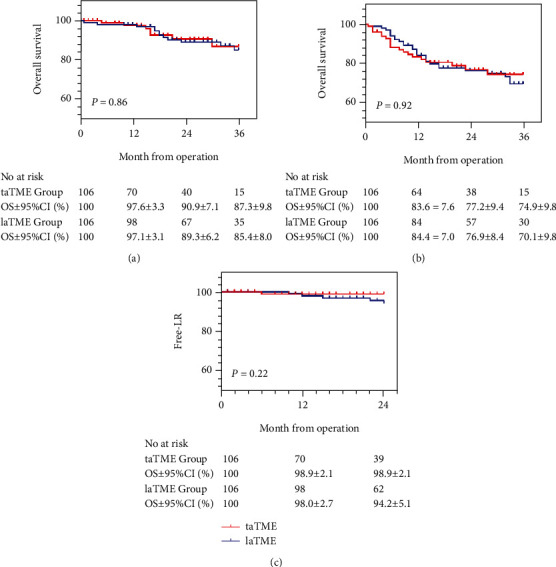
Kaplan–Meier plots of overall survival (a). Kaplan–Meier plots of disease-free survival (b). Kaplan–Meier plots of free-local recurrent (c).

The median follow-up time of the taTME and laTME patients was 21.80 ± 18.153 (1-121) months and 30.29 ± 13.439 (1-73) months, respectively. As shown in Figures 3(a) and 3(b), there was no significant difference between the two groups for 3-year OS (87.3% VS 85.4%, *P* = 0.86) or 3-year DFS (74.9% vs 70.1%, *P* = 0.92). The 2-year cumulative local recurrence rate (Figure 3(c)) was similar between the two groups (1.1% vs 5.8%, *P* = 0.22). Within 2 years after surgery, in the taTME group, one patient had a local recurrence 5 months after the operation shortly, and the patient just received palliative chemotherapy, at last. In the laTME group, 5 patients presented local recurrence at 10, 12, 15, 22, and 24 months after surgery, respectively, within the 2-year follow-up period.

## 4. Discussion

It remains difficult worldwide to achieve a high standard of TME to treat low rectal cancer male patients that underwent neoadjuvant therapy [2, 17]. The taTME procedure has been considered to have the potential to solve this problem [7]. In this study, we compared the performance of taTME and laTME in “challenging” patients. Results showed that taTME could reduce pelvis operative time and intraoperative blood loss. Furthermore, the number of patients with protective loop ileostomy and postoperative complications proved to be significantly lower in the taTME group than in the laTME group. Most importantly, taTME also showed potential in achieving high-quality resected specimens and great long-term results in “challenging” patients.

In this study, the pelvic operative time of taTME was shorter than that of laTME, and the intraoperative blood loss was also reduced compared with laTME. This may reflect that laparoscopic surgery still fails to solve the exposure problem in pelvic surgery for “challenging” patients, as it is too difficult to perform complex operations in the deep pelvis with rigid straight laparoscopic instruments [18–20], and it is also hard to obtain adequate traction of the rectum [21]. Moreover, patients after neoadjuvant therapy are generally prone to bleeding, resulting in the declined clarity of visual field during the operation, thus making the procedure even more “challenging”. In contrast, the visual surgical field of taTME is clearer due to the changed surgical approach, and the operative space is relatively larger [22].

Regarding postoperative complications, for any new surgical method or approach, the incidence of complications should be controlled within the acceptable range of patients. The previously published preliminary results of an RCT, led by our center, showed an 8.1% incidence of anastomotic leakage for taTME [23]. Some other RCTs, such as COLOR II and CLASICC trials, reported that the incidence of anastomotic leakage in laTME was 13% and 10%, respectively. In this study, both groups showed a higher AL incidence rate since “challenging” patients may be at high risk of AL [24–29]. In males, it is considered that the pelvis tends to be longer, the sacrum is more curved, and the subpubic arch is narrower [8]. Furthermore, the male pelvic entrance is significantly narrower than that of the female [22]. Nevertheless, the incidence of anastomotic leakage in the taTME group was significantly lower compared with the laTME group (14.2% vs 25.2%, *P* = 0.015), which is a rather remarkable finding. The reason for this result may be that taTME can retain more rectum. At the same time, because of the convenience of transanal operation, the anastomosis was strengthened routinely by 3-0 vicryl (Ethicon, Somerville, NJ, United States), after using the stapler. It is also worth noting that there were fewer patients with C-D grade III in the taTME group, which also reflects the improved safety of taTME surgery. In addition, taTME has a lower protective loop ileostomy rate (70.8% vs 92.5%, *P* < 0.001), which can help to avoid a secondary operation. This is good news for patients both in terms of quality of life and postoperative recovery. Concerning other postoperative complications, the incidence of taTME was also lower than that of laTME, although the difference was not statistically significant. However, this trend explains why taTME leads to more speedy postoperative recovery; the median postoperative hospital stay for taTME was one day shorter than that for laTME. It is worth noting that according to the results of our previous report about the taTME learning curve [30], there are still individual surgeons in the taTME group who are impacted by the learning curve. However, compared with the laTME group, it still showed good postoperative results. Therefore, the prospects of implementing taTME for the treatment of “challenging” patients are highly promising.

In the literature, the male gender has been acknowledged as a clinical variable correlated with difficult pelvic dissection and incomplete mesorectal excision, or positive CRM after laTME [31]. Several reports have shown that taTME is helpful to obtain high-quality specimens and lower rates of positive DRM and CRM, which can affect the patient survival and prognosis outlook [32]. This study could not demonstrate a significant difference in pathological outcomes between the two groups, although the positive CRM rate for the taTME group was lower than that for the laTME group (0% vs 1.9%). While the mesorectal quality was complete or near complete in both groups, the number of complete cases was higher in the taTME group. The above results are consistent with those of previous studies. In contrast to earlier findings [33, 34], DRM length was shorter in the taTME group than the laTME group, although the difference was statistically insignificant. This may be explained by the fact that, in the taTME group, while keeping a sufficient safety resection margin, a longer rectum can be retained to reconstruct the continuity of the intestine for anus preservation.

When the Norwegian taTME collaborative group reported the results of their study on the local recurrence rate of taTME, it caused a huge controversy. Significantly higher local recurrence rates were obtained with taTME in 157 patients compared to the national average (7.6% vs 2.4%) [35]. In this study, the 2-year cumulative local recurrence rate was smaller in the taTME group as compared with the laTME group. Nonetheless, the difference did not reach statistical significance (1.1% vs 5.8%, *P* = 0.22). These results indicate that the taTME may potentially reduce local recurrence rates with accurate DRM positioning and reducing tumor blocking effect, which shows its advantages for “challenging” patients. Although the rate of DFS and OS in the taTME group was higher, this study has not shown significant differences between the two groups. In fact, all of taTME, laTME, and open surgery TME are performed under the principles of TME. As long as the surgery is of high quality and obtains good histopathological results, long-term postoperative oncology outcomes, in theory, should be similar among these approaches with no significant difference. This has been proved by many studies, including some RCTs and meta-analyses [2, 36, 37]. Consequently, taTME may improve the long-term survival of “challenging” patients by higher quality TME.

Certain limitations of this study need to be acknowledged. First, despite using PSM to decrease the selection bias, residual confounding may remain from other unmatched variables. Meanwhile, the remaining number of taTME at 24 months is only 39 out of 106. A large-scale RCT would be needed to prove the desirable effects of taTME. The long-term and functional results of an ongoing TaLaR trial will be due in 2021, which are much anticipated. Secondly, the sample size was small, which might increase the chance of type II errors. Furthermore, this study was based on a single center. Thus, results may not be representative. Another factor that may cause bias is the lower average BMI score for Asian patients than occidental patients, so high BMI was not included in this study's definition of “challenging” patients.

## 5. Conclusions

The present study demonstrated that taTME might reduce the incidence of postoperative complications, especially of anastomotic leakage in these “challenging” patients. The lower rate of protective loop ileostomy subsequently reduces the blow to the patient's second operation. Thus, taTME may be considered to have clear advantages for “challenging” patients.

## Figures and Tables

**Table 1:** Demographic and clinical data before and after propensity score matching.

Characteristics	Before propensity score matching		*P*	After propensity score matching		*P*
	taTME (*n* = 112)	laTME (*n* = 213)		taTME (*n* = 106)	laTME (*n* = 106)	
Age, mean ± SD (range)	56 ± 12 (23-78)	53 ± 12 (20-80)	0.08	55 ± 12 (23-78)	56 ± 12 (26-79)	0.80
BMI, mean ± SD (range)	23.0 ± 2.9 (17.1-37.6)	22.5 ± 3.0 (15.9-34.2)	0.10	23.0 ± 2.9 (17.2-32.3)	22.9 ± 3.2 (16.9-34.3)	0.85
Tumor location, mean ± SD (range)	3.5 ± 0.9 (1.0-5.0)	3.9 ± 0.9 (1.4-5.0)	<0.001	3.6 ± 0.9 (2.0-5.0)	3.8 ± 0.9 (1.4-5.0)	0.20
Tumor size (cm), mean ± SD (range)	3.0 ± 1.3 (0.3-6.6)	2.7 ± 1.7 (0-8.0)	0.07	3.0 ± 1.3 (0.3-6.6)	2.8 ± 2.0 (0-8.0)	0.20
Preoperative intestinal obstruction, *n* (%)			<0.001			—
None	112 (100%)	199 (93.4%)		106 (100%)	106 (100%)	
Incomplete	0 (0%)	14 (6.6%)		—	—	
History of past abdominal surgery, *n* (%)			0.68			1.00
Without	105 (93.8%)	202 (94.8%)		101 (95.3%)	102 (96.2%)	
With	7 (6.3%)	11 (5.2%)		5 (4.7%)	4 (3.8%)	
cT, *n* (%)			0.23			0.53
T0	0 (0%)	1 (0.5%)		—	—	
T1	1 (0.9%)	1 (0.5%)		1 (0.9%)	0 (0%)	
T2	12 (10.7%)	41 (19.2%)		12 (11.3%)	18 (17.0%)	
T3	90 (80.4%)	156 (73.2%)		86 (81.1%)	82 (77.4%)	
T4	9 (8%)	14 (6.6%)		7 (6.6%)	6 (5.7%)	
cN, *n* (%)			0.09			0.63
N0	63 (56.3%)	145 (68.1%)		60 (56.6%)	66 (62.3%)	
N1	33 (29.5%)	47 (22.1%)		31 (29.2%)	25 (23.6%)	
N2	16 (14.3%)	21 (9.9%)		15 (14.2%)	15 (14.2%)	
cM, *n* (%)			0.24			1.00
M0	108 (96.4%)	210 (98.6%)		102 (96.2%)	103 (97.2%)	
M1	4 (3.6%)	3 (1.4%)		4 (3.8%)	3 (2.8%)	
Type of neoadjuvant therapy regimen, *n* (%)			<0.001			0.47
Only chemotherapy	77 (68.8%)	89 (41.8%)		71 (67.0%)	66 (62.3%)	
Radiochemotheraoy	35 (31.3%)	121 (56.8%)		35 (33.0%)	40 (37.7%)	
Only radiotherapy	0(0%)	3(1.4%)		—	—	

**Table 2:** Perioperative outcomes.

Characteristics	Group		*P*
	taTME (*n* = 106)	laTME (*n* = 106)	
Total operative time minutes(min) mean ± SD (range)	225.0 ± 81.5 (102-600)	241.1 ± 88.6 (105-586)	0.425
Pelvic operative time minutes(min) mean ± SD (range)	62.2 ± 14.2 (43-98)	81.1 ± 18.9 (55-178)	0.003
Intraoperative blood loss(mL), mean ± SD (range)	90 ± 156 (10-1500)	103 ± 136 (20-1300)	0.004
Blood transfusion, *n* (%)			1
Yes	2 (98.1%)	2 (98.1%)	
No	104 (1.9%)	104 (1.9%)	
Distance from anastomosis to anal margin(cm), mean ± SD (range)	2.3 ± 1.0 (0-5)	2.0 ± 1.1 (0-5)	0.051
Protective loop ileostomy, *n* (%)			<0.001
Yes	75 (70.8%)	98 (92.5%)	
No	31 (29.2%)	8 (7.5%)	
Conversion, *n* (%)			0.246
Yes	—	3 (2.8%)	
No	106 (100.0%)	103 (97.2%)	
Postoperative transfer to ICU, *n* (%)			1
Yes	3 (2.8%)	3 (2.8%)	
No	103 (97.2%)	103 (97.2%)	
Postoperative complication, *n* (%)			0.003
Yes	21 (19.8%)	41 (38.7%)	
No	85 (80.2%)	65 (61.3%)	
Types of complications, *n* (%)			
Anastomotic leakage	15 (14.2%)	27 (25.2%)	0.017
Postoperative ileus	2 (0.9%)	5 (4.7%)	0.445
Anastomotic stenosis	5 (4.7%)	5 (4.7%)	1
Postoperative abdominal hemorrhage	—	1 (0.9%)	
Rectourethral fistula	1 (0.9%)	2 (0.9%)	1
Urinary retention	2 (0.9%)	6 (5.7%)	0.28
Pulmonary infections	—	1 (0.9%)	
Pelvic abscess	—	1 (0.9%)	
Anastomotic ischemic enteritis	—	1 (0.9%)	
Clavien-Dindo grade, *n* (%)^a^			0.03
I/II	14 (66.7%)	30 (73.2%)	0.593
III	7 (33.3%)	9 (22.0%)	0.332
IV	—	1 (2.4%)	
V	—	1 (2.4%)	
Postoperative hospital stay (days), median (range)	9 (3-48)	10 (6-30)	<0.001
30-day postoperative mortality, *n* (%)			1
Yes	—	1 (0.9%)	
No	106 (100.0%)	105 (99.1%)	

^a^Proportion of patients with postoperative complications in each group: 21 (taTME group) and 41 (laTME group).

**Table 3:** Pathological outcomes.

Characteristics	Group		*P*
	taTME(*n* = 106)	laTME(*n* = 106)	
Length of resected intestine in centimeters (cm), mean ± SD (range)	12.6 ± 4.7 (7.0-34.0)	13.1 ± 5.1 (6.5-36.0)	0.458
Quality of the mesorectum specimen			0.269
Complete	101 (95.3%)	97 (91.5%)	
Nearly complete	5 (4.7%)	9 (8.5%)	
Distance between tumor and distal resection margin in cm, mean ± SD (range)	1.2 ± 0.9 (0.1-4.5)	1.3 ± 1.9 (0.2-4.0)	0.394
Number of harvested lymph nodes, median (range)	13 (1-38)	12 (0-38)	0.285
DRM status, *n* (%)			1
Negative	105 (99.1%)	105 (99.1%)	
Positive	1 (0.9%)	1 (0.9%)	
Lymphovascular invasion, *n* (%)			0.683
Negative	102 (96.2%)	104 (98.1%)	
Positive	4 (3.8%)	2 (5.7%)	
Perineural invasion, *n* (%)			0.422
Negative	97 (91.5%)	100 (94.3%)	
Positive	9 (8.5%)	6 (5.7%)	
CRM status, *n* (%)			0.498
Negative	106 (100%)	104 (98.1%)	
Positive	0 (0%)	2 (1.9%)	
pT, *n* (%)			0.074
PCR	16 (15.1%)	24 (22.6%)	
T1	3 (2.8%)	8 (7.5%)	
T2	19 (17.9%)	21 (19.8%)	
T3	67 (63.2%)	49 (46.2%)	
T4	1 (0.9%)	4 (3.8%)	
pN, *n* (%)			0.17
N0	77 (72.6%)	79 (74.5%)	
N1	17 (16%)	22 (20.8%)	
N2	12 (11.3%)	5 (4.7%)	
pM, *n* (%)			0.7213
M0	101 (95.3%)	103 (97.2%)	
M1	5 (4.7%)	3 (2.8%)	

## Data Availability

The data used to support the findings of this study are available from the corresponding author upon request.

## References

[1] Heald R. J. (1979). A new approach to rectal cancer. *British Journal of Hospital Medicine*.

[2] van der Pas M. H. G. M., Haglind E., Cuesta M. A. (2013). Laparoscopic versus open surgery for rectal cancer (COLOR II): short-term outcomes of a randomised, phase 3 trial. *The Lancet Oncology*.

[3] Kang S. B., Park J. W., Jeong S. Y. (2010). Open versus laparoscopic surgery for mid or low rectal cancer after neoadjuvant chemoradiotherapy (COREAN trial): short-term outcomes of an open- label randomised controlled trial. *The Lancet Oncology*.

[4] Velthuis S., Nieuwenhuis D. H., Ruijter T. E. G., Cuesta M. A., Bonjer H. J., Sietses C. (2014). Transanal versus traditional laparoscopic total mesorectal excision for rectal carcinoma. *Surgical Endoscopy*.

[5] Sylla P., Rattner D. W., Delgado S., Lacy A. M. (2010). NOTES transanal rectal cancer resection using transanal endoscopic microsurgery and laparoscopic assistance. *Surgical Endoscopy*.

[6] Velthuis S., Van Den Boezem P. B., Van Der Peet D. L., Cuesta M. A., Sietses C. (2013). Feasibility study of transanal total mesorectal excision. *The British Journal of Surgery*.

[7] Heald R. J. (2013). A New Solution to some Old Problems: Transanal TME. *Techniques in Coloproctology*.

[8] Lewis C. L., Laudicina N. M., Khuu A., Loverro K. L. (2017). The human pelvis: variation in structure and function during gait. *The Anatomical Record*.

[9] Bosch S. L., Nagtegaal I. D. (2012). The importance of the pathologist’s role in assessment of the quality of the mesorectum. *Current Colorectal Cancer Reports*.

[10] Austin P. C. (2011). An introduction to propensity score methods for reducing the effects of confounding in observational studies. *Multivariate Behavioral Research*.

[11] Heinze G., Jüni P. (2011). An Overview of the Objectives of and the Approaches to Propensity Score Analyses. *European Heart Journal*.

[12] Clavien P. A., Barkun J., De Oliveira M. L. (2009). The Clavien-Dindo Classification of Surgical Complications: Five-Year Experience. *Annals of Surgery*.

[13] American Joint Committee on Cancer (2010). *Cancer Staging Manual*.

[14] Quirke P., Dixon M. F., Durdey P., Williams N. S. (1986). Local recurrence of rectal adenocarcinoma due to inadequate surgical resection: histopathological study of lateral tumour spread and surgical excision. *Lancet*.

[15] Nagtegaal I. D., Van de Velde C. J. H., Van Der Worp E., Kapiteijn E., Quirke P., van Krieken J. H. J. (2002). Macroscopic evaluation of rectal cancer resection specimen: clinical significance of the pathologist in quality control. *Journal of Clinical Oncology*.

[16] Brierley J. D., Gospodarowicz M. K., Wittekind C. (2017). *TNM Classification of Malignant Tumours*.

[17] Smith J. J., Garcia-Aguilar J. (2015). Advances and Challenges in Treatment of Locally Advanced Rectal Cancer. *Journal of Clinical Oncology*.

[18] Dayal S., Battersby N., Cecil T. (2017). Evolution of surgical treatment for rectal cancer: a review. *Journal of Gastrointestinal Surgery*.

[19] Grass J. K., Perez D. R., Izbicki J. R., Reeh M. (2019). Systematic review analysis of robotic and transanal approaches in TME surgery- a systematic review of the current literature in regard to challenges in rectal cancer surgery. *European Journal of Surgical Oncology*.

[20] Guillou P. J., Quirke P., Thorpe H. (2005). Short-term endpoints of conventional versus laparoscopic-assisted surgery in patients with colorectal cancer (MRC CLASICC trial): multicentre, randomised controlled trial. *Lancet*.

[21] Persiani R., Biondi A., Pennestrì F. (2018). Transanal total mesorectal excision vs laparoscopic total mesorectal excision in the treatment of low and middle rectal cancer: a propensity score matching analysis. *Diseases of the Colon and Rectum*.

[22] Lorenzon L., Bini F., Landolfi F. (2021). 3D pelvimetry and biometric measurements: a surgical perspective for colorectal resections. *International Journal of Colorectal Disease*.

[23] Kang L., Chen Y. G., Zhang H. (2020). Transanal total mesorectal excision for rectal cancer: a multicentric cohort study. *Gastroenterology Report*.

[24] Akiyoshi T., Kuroyanagi H., Oya M. (2009). Factors affecting the difficulty of laparoscopic total mesorectal excision with double stapling technique anastomosis for low rectal cancer. *Surgery*.

[25] Zhou X. C., Su M., Hu K. Q. (2016). CT pelvimetry and clinicopathological parameters in evaluation of the technical difficulties in performing open rectal surgery for mid-low rectal cancer. *Oncology Letters*.

[26] Kim J. Y., Kim Y. W., Kim N. K. (2011). Pelvic anatomy as a factor in laparoscopic rectal surgery: a prospective study. *Surgical Laparoscopy Endoscopy & Percutaneous Techniques*.

[27] Zhou X., Su M., Hu K. (2015). Applications of computed tomography pelvimetry and clinical-pathological parameters in sphincter preservation of mid-low rectal cancer. *International Journal of Clinical and Experimental Medicine*.

[28] Hottenrott C. (2011). Predicting and preventing anastomotic leakage after low anterior resection for rectal cancer. *World Journal of Surgery*.

[29] Kobayashi M., Mohri Y., Ohi M. (2014). Risk factors for anastomotic leakage and favorable antimicrobial treatment as empirical therapy for intra-abdominal infection in patients undergoing colorectal surgery. *Surgery Today*.

[30] Zeng Z., Liu Z., Huang L. (2021). Transanal Total Mesorectal Excision in Mid-Low Rectal Cancer: Evaluation of the Learning Curve and Comparison of Short-term Results With Standard Laparoscopic Total Mesorectal Excision. *Diseases of the Colon & Rectum*.

[31] Penna M., Hompes R., Arnold S. (2017). Transanal total mesorectal excision. *Annals of Surgery*.

[32] Kitz J., Fokas E., Beissbarth T. (2018). Association of plane of total mesorectal excision with prognosis of rectal cancer secondary analysis of the CAO/ARO/AIO-04 phase 3 randomized clinical trial. *JAMA Surgery*.

[33] Ong G. K., Tsai B., Patron R. L. (2021). Transanal total mesorectal excision achieves equivalent oncologic resection compared to laparoscopic approach, but with functional consequences. *American Journal of Surgery*.

[34] Zeng Z., Luo S., Chen J., Cai Y., Zhang X., Kang L. (2020). Comparison of pathological outcomes after transanal versus laparoscopic total mesorectal excision: a prospective study using data from randomized control trial. *Surgical Endoscopy*.

[35] Wasmuth H. H., Færden A. E., Myklebust T. Å. (2019). Transanal total mesorectal excision for rectal cancer has been suspended in Norway. *British Journal of Surgery*.

[36] Jeong S. Y., Park J. W., Nam B. H. (2014). Open versus laparoscopic surgery for mid-rectal or low-rectal cancer after neoadjuvant chemoradiotherapy (COREAN trial): survival outcomes of an open- label, non-inferiority, randomised controlled trial. *The Lancet Oncology*.

[37] Denost Q., Loughlin P., Chevalier R., Celerier B., Didailler R., Rullier E. (2018). Transanal versus abdominal low rectal dissection for rectal cancer: long-term results of the Bordeaux’ randomized trial. *Surgical Endoscopy*.

